# Quality and Stability Equivalence of High Pressure and/or Thermal Treatments in Peach–Strawberry Puree. A Multicriteria Study

**DOI:** 10.3390/foods10112580

**Published:** 2021-10-26

**Authors:** Iulia Bleoanca, Livia Patrașcu, Daniela Borda

**Affiliations:** 1 Faculty of Food Science and Engineering, Dunarea de Jos University of Galati, 111 Domneasca Str., 800201 Galati, Romania; Iulia.Bleoanca@ugal.ro; 2 Cross-Border Faculty, Dunarea de Jos University of Galati, 111 Domneasca Str., 800201 Galati, Romania; Livia.Mantoc@ugal.ro

**Keywords:** peaches, strawberries, high pressure, thermal treatment, equivalent effects, refrigerated storage

## Abstract

A bottom-up approach identifying equivalent effects of high-pressure processing (HPP—600 MPa, 20 °C, 10 min), thermal treatment (TT—70 °C, 15 min) and high pressure-mild thermal processing (HPMT—600 MPa, 50 °C, 10 min) on quality and stability of peach–strawberry puree was applied during refrigerated storage. TT and HPP ensured 3-log aerobic bacteria inactivation at first, while HPMT reduction was below the detection limit. After 21 days all samples had equivalent microbiological stability. A 2.6-fold increase in the residual activity of PPO and POD was found in the HPP sample compared to TT and HPMT samples (1st day); after 21 days PPO, POD and TPC were equivalent for TT and HPP peach–strawberry purees. Equivalent volatile profile and rheology behavior was observed after 21 days of all samples’ storage. Meanwhile, the color of the HPP, TT and HMPT samples remained significantly different (*p* < 0.05) throughout the whole storage period, with the lowest browning index registered for HPP samples.

## 1. Introduction

Fruit purees are consumed alone or as ingredients in many ready-to-eat healthy and nutritious deserts such as jams or fruit ice creams, yogurts or smoothies and mostly intended for children and the elderly. Moreover, the joint global strategy on diet and health of World Health Organization and Food and Agriculture Organization underlines the importance of consumption of at least 400 g fruits and vegetables per day as a major component of a balanced, high-quality diet [1].

Nevertheless, fruit purees have a limited storage period. Fruit spoilage occurs naturally as a result of an interplay between physiological (enzymatic and metabolic activity of the fruit tissue) and microbiological deterioration phenomena (spoilage, microorganism growth) [2].

Fruit purees’ shelf-life can be extended by traditional thermal treatment (TT), which usually determines undesirable appearance changes, flavor and nutritional losses of the final product or by alternative treatments, such as high pressure processing (HPP), which proved to be a successful commercial solution with minimal negative effects on the overall quality of liquid or solid foods [3,4]. Nevertheless, application of HPP alone is not a magic bullet, as it usually does not completely inactivate the activity of browning related enzymes mainly responsible for deleterious effects in fruits: no peroxidase (POD) inactivation at pressures lower than 800 MPa in Reineta apple slices [5], 40% POD inactivation in strawberries of three cultivars after HP treatment at 600 MPa, 20 °C, for 5 min [6], 26% POD inactivation in pears treated at 600 MPa, 20 °C, for 5 min [7]; 35% inactivation of polyphenoloxidase (PPO) in peach puree after HPP at 600 MPa, 25 °C for 5 min [8], 70% PPO inactivation in pear slices after 600 MPa, 20 °C for 5 min [7], and only 7% PPO inactivation in apple, orange, strawberry and banana smoothie after 600 MPa, at 10 °C, for 3 min [9]. Limited information is available on fruit spoilage yeasts and fungi survival and growth after HPP treatment [10].

The use of combined mild thermal treatment with HPP (HPMT) could be a solution for extending fruit purees shelf-life that maintains the benefits of HPP, improve the storage stability of processed horticultural products while avoiding the flavor and nutritional losses conventionally associated with high intensity thermal processes [7]. Moreover, blending fruits represent a convenient and cost-efficient way of including two or more fruits into the food product and benefit from the presence of high-value fruits with reduced shelf life such as strawberries and by more popular medium-value ones, such as peaches.

The aim of the present study was to assess the peach–strawberry puree changes after HPP, TT and HPMT considering multiple microbiological and physicochemical factors correlated with quality deterioration and microbial stability during refrigerated storage. However, comparison of the effects of different processes on microbial, quality, and shelf-life of food products should be discussed from the perspective of equivalent treatments that provide an equivalent effect [11,12]. This paper discusses the equivalence of HPP, TT and HPMT treatments selected considering the multi-quality and stability criteria.

## 2. Materials and Methods

### 2.1. Sample Preparation

Peaches (*Prunus persica*, Redhaven cultivar, Constanta, Romania) and strawberries (*Fragaria ananassa**,*** Premial cultivar, Timpurii de Pitesti, Romania) were bought form the local market in Galati, Romania. Fruits were in an adequate state of commercial maturity, without any bruises or damages. The peaches and strawberries were washed with cold water and, while the peaches were peeled, sliced and the kernel was removed, the calyx of the strawberries was detached. Immediately after, the 1:1 (*w*/*w*) peaches and strawberries mixture was homogenized with a domestic blender (Philips HR7761/00), for 180 s at maximum velocity and quickly transferred to a food grade pouch PET/EVM (combination of biaxially oriented polyester with coextruded barrier film of the structure polyethylene/EVOH/m-polyethylene) (2.5 × 2.5 cm^2^, thickness 80 µm) and immersed into iced water to prevent browning. Further, the pouches containing 10 g of sample mixture were vacuumed, thermally sealed, and kept at 4 °C in darkness until further use [9].

#### 2.1.1. Thermal Processing

The pouches with fruit mixture were incubated in a thermostatic water bath at 70 °C for 15 min. Timing was started after the temperature was equilibrated at 70 °C in the sample, as measured by type K thermocouple in one control pouch. The come-up time during the thermal treatment was 3.5 ± 0.4 min. Immediately after TT, the samples were cooled in iced water to stop the thermal effect and further stored at 4 °C.

#### 2.1.2. Combined Thermal and/or High-Pressure Treatment

The pressure treatments were conducted in a laboratory-scale multivessel (four vessels, each of 100 mL capacity) high-pressure equipment (Resato, Roden, The Netherlands, 2011). As a pressure transmitting fluid a mixture of water and propylene glycol (TR15, Resato) was used. Two types of pressure treatments were performed: HPP at 600 MPa and 20 °C (prior processing the samples and the high pressure vessels were pre-equilibrated at 20 °C); HPMT at 600 MPa and 50 °C (temperature of the compression fluid was set and the vessels were pre-equilibrated at 50 °C; the pouches were preheated at 50 °C in a thermostatic water bath to attain the target processing temperature before HPMT to reduce the temperature differences between samples and environment temperature).

The compression rate was 200 MPa/min, until the preset pressure was reached. After the come-up time, a supplementary 1 min equilibration time was considered in all experiments. During the HPP treatment, the adiabatic heating increased temperature in the surrounding liquid from 20 ± 2.3 °C to almost 37 ± 3.4 °C while in HPMT treatment the temperature varied from 50 ± 3 °C to 70 ± 2.9 °C. The adiabatic heating profiles during pressure increase for the HPP and HPMT treatment are presented in Supplementary File Figure S1.

The temperature of the samples’ surrounding environment was monitored throughout the treatments with thermocouples placed inside the pressure vessels. After the time set for HPP and HPMT experiments (10 min) the samples were decompressed in less than 10 s and placed in a cooled iced water bath to stop further reactions and later stored in refrigeration conditions at 4 °C. The analyses performed in the first day were done after approximately one 12 h of refrigerated storage. Each treatment was repeated three times.

### 2.2. Microbial Enumeration

Microbial quality of the peach–strawberry puree was assessed through mesophilic aerobic bacteria (MAB), molds and yeasts (M&Y). Serial decimal dilutions of 1 g peach–strawberry puree prepared in sterile 0.1% saline solution were plated on Plate Count Agar (BD Difco, Franklin Lakes, NJ, USA) for MAB and on Malt Extract Agar (Merck, Bucharest, Romania) for M&Y. Following incubation of MAB plates at 37 °C for 48 h, of M&Y plates at 25 °C for 5 days, the results were reported as logarithm of colony forming units per gram (Log CFU g^−1^) [13]. All samples were analyzed in triplicate.

### 2.3. pH and Soluble Solids

pH was determined at room temperature with pH meter (S230 Mettler Toledo, Zaventem, Belgium). The pH was measured twice for all samples, in the beginning, after 14 and 21 days of refrigerated storage.

### 2.4. Enzyme Extraction

The refrigerated peach–strawberry puree samples of approximately 10 g were unpacked and extracted in 20 mL McIlvaine buffer (1:2 *w*/*v*) for polyphenol oxidase (PPO) assay or in 20 mL 0.2 M phosphate buffer pH 6.6 for peroxidase (POD) assay. Two steps of centrifugation (6000× *g*) were applied at 4 °C to clarify the extracts. The supernatant was further used as crude enzyme extract for PPO, respectively POD activity determination. All experiments were conducted in triplicate. All the reagents used in the extraction and measurement were of analytic grade or high degree of purity.

#### 2.4.1. PPO Activity Assay

The PPO activity was determined according to the method described by [8]. Briefly, to determine PPO activity, 0.350 mL of extract was mixed in 1.420 mL McIlvaine buffer (pH 6.6) and 0.730 mL 0.175 M pyrocatechol was added. The absorbance increase was measured spectrophotometrically (UV VIS Cintra 202, Braeside, Australia) at 25 °C and 420 nm for 10 min and the slope of the linear curve of absorbance versus time was considered the enzyme activity. For the blank sample no extract was added into the reaction mixture. The residual activity was reported to the control sample with no treatment applied, measured in the first day of storage, using Equation (1) as suggested by [14]:

(1) $$\mathrm{RA}=\frac{\mathrm{Enzyme}\;\mathrm{activity}\;\mathrm{in}\;\mathrm{the}\;\mathrm{sample}}{\mathrm{Enzyme}\;\mathrm{activity}\;\mathrm{in}\;\mathrm{the}\;\mathrm{control}\;\mathrm{sample}}\times 100\;\left(\%\right)$$

#### 2.4.2. POD Activity Assay

The reaction mixture consisted of 0.1 mL enzymatic extract in 0.2 M phosphate buffer pH 6.6, 0.2 mL 0.09 M p-phenylenediamine, 0.1 mL of 0.5 M hydrogen peroxide and 2.7 mL of 0.2 M phosphate buffer, pH 6.6. The absorbance was measured in a kinetic mode for 5 min at 485 nm and 25 °C against blank sample using a spectrophotometer (UV VIS Cintra 202, Braeside, Australia). The blank sample contained the same components, but instead of 0.1 mL enzymatic extract in 0.2 M phosphate buffer pH 6.6, 0.1 mL of 0.2 M phosphate buffer, pH 6.6, was added. The enzyme activity of the control sample was considered the activity measured in the first day of storage with no treatment applied. The change of absorbance/min was reported as enzyme activity. The residual POD activity was determined using the same formula as for PPO activity (Equation (1)).

#### 2.4.3. DPPH Radical Scavenging Activity

The DPPH radical scavenging assay was determined according to [15] with some small changes. Briefly, 0.125 mM DPPH solution in methanol was prepared fresh daily. An aliquot of 0.1 mL of fruit extract in phosphate buffer was mixed with 0.9 mL of 0.1 M Tris/HCl, pH 7.4 and 2 mL of 0.125 mM DPPH solution. The mixture was shaken vigorously and stored for 15 min in dark conditions at approximately 25 °C. The absorbance of the reaction mixture was then measured at 515 nm and 25 °C using a UV VIS Cintra 202 spectrophotometer. All measurements were done in triplicate.

(2) $$\%\mathrm{AA}=\frac{A_{i}-A_{t}}{A_{i}}\times 100$$

where AA is the percentage of the antiradical activity; A_i_ is the absorbance at 517 nm of the control (no added sample) and A_t_ is the absorbance in presence of fruit extract after 15 min.

#### 2.4.4. Total Phenolic Content (TPC)

Total phenolic contents (TPC) in phosphate peach–strawberry puree extracts were determined according the modified Folin–Ciocalteu colorimetric method as described by [15] with small changes. Briefly, TPC was spectrophotometrically evaluated at 765 nm. Firstly, 0.3 mL of Folin–Ciocalteu reagent was added to 0.1 mL sample. The sample was incubated for 1 min at 37 °C and further 2.4 mL of sodium carbonate solution 7.5% (*w*/*w*) was added to the mixture and incubated for 15 min at 37 °C. The average value of triplicate measurements was expressed as Gallic acid equivalents, in mg per 100 g fresh fruit weight (mg GAE/100 g FW) using a Gallic acid standard curve.

### 2.5. Rheology

Rheometric measurements were performed with a control stress AR 2000ex rheometer (TA Instruments, New Castle, DE, USA), using a plate-plate geometry with 60 mm in diameter and a gap of 1 mm. The temperature was maintained at 20 °C by the Peltier system. During experiments, the measurement system was covered with a special cover plate device to avoid water evaporation. Additionally, 1 mL of water was added in the geometry solvent trap. Rheological behavior of samples was observed during steady and then forced flow conditions, by maintaining first the shear rate at a value of 10 s^−1^ for 5 min, then increasing it up to 100 s^−1^. Apparent viscosity was considered as a function of shear rate and time.

### 2.6. SMPE Gas Chromatography-Mass Spectrometric (GC-MS) Analysis

The untargeted fingerprints of the volatiles from the peach–strawberry puree samples were analyzed using a Trace GC-MS Ultra equipment with SPME extraction system, and the separated volatile compounds were detected with a ionic trap-MS ITQ 900 (Thermo Scientific, Waltham, MA, USA). The method of incubation, extraction and analysis was optimized, beforehand. The column used was TG-WAX capillary column (60 m × 0.25 mm, i.d. 0.25 μm). Helium (99.996% purity, Messer Gaz S.R.L., Bucharest, Romania) was used as a carrier gas at a flow of 1 mL/min. Samples of 5 g peach–strawberry puree were weighed in glass vials (20 mL) with 1 g of saturated (NH_4_)_2_SO_4_ (Redox SRL, Bucharest, Romania) further spiked with 5 μL 2-octanol 0.651 g/mL (Sigma Aldrich Chemie GmbH, Steinheim, Germany) added as internal standard. Then, the vials were sealed, and the mixture was maintained at 45 °C for 10 min for equilibration before concentration by SPME on a CAR/PDMS fiber. The extraction of the volatiles under isothermal conditions at 45 °C was made over 25 min followed by 7 min of desorption into the GC injection port. The temperature ramp selected for the analysis was 45 °C isothermal treatment for 3 min followed by an increase to 50 °C at 5 °C/min and to 100 °C with 8 °C/min, to 150 °C at 10 °C/min and finally to 230 °C at 12 °C/min, when temperature was kept constant for 2 min. After 2 min at the final temperature (230 °C), the oven was cooled again to the initial temperature. The temperature of the transfer line in MS was set to 270 °C. Mass spectra were obtained from the full scan of the positive ions resulted with a scanning in the 50 to 650 *m*/*z* range and operated with an electron impact (EI)-mode of 200 eV. The compounds were tentatively identified in comparison with the mass spectra from Wiley and Nist 08 library database available with Xcalibur software. Each analysis was performed in triplicate, in the first and the 21st day of storage. The retention indices (RI) of each compound were calculated by using n-alkane series from C8-C40 (Sigma Aldrich Chemie GmbH, Steinheim, Germany) under the same conditions. The volatile organic compounds (VOCs) were estimated semi-quantitatively using 2-octanol as internal standard (IS) and Equation (3) [16,17]:

(3) VOCconc= ISconc×VOCpeak areaISpeak area

where VOC_peak area_ is the area of the integrated individual peak, IS_peak area_ is the area of 2-octanol in the spiked samples and IS_conc_ is the concentration of internal standard (2-octanol).

### 2.7. Color Attributes

The color changes of the analyzed peach–strawberry purees (L*, a*, b*) were evaluated immediately after processing, after 14 and 21 days of storage at refrigeration temperature (4 °C) using a Hunter Lab MiniScan colorimeter (Hunter Associates Laboratory, Inc., Reston, VA, USA), with the light source set on D65 and an observation angle of 10°. The CIELAB L* coordinate approximating sample luminosity (0—black, 100—white), a* indicating the difference in red (positive a* values) and green (negative a* values) and b* showing the difference in yellow (positive b* values) and blue (negative b* values) were evaluated. Chroma (C*), calculated according to Equation (4), was used to determine the color saturation of samples perceived by the human eye through comparison of transition from grey (low C* values) to the pure color (high C* values) [18].

(4) $$C^{*}=\sqrt{\left(a^{2}+b^{2}\right)}$$

Hue angle (h°) designating the relative amount of redness (0° or 360°), yellowness (90°), greenness (180°) or blueness (270°) was calculated using Equation (5) [18]:

h° = tan^−1^(b*/a*) (5)

The total color difference ΔE* was determined with Equation (6) by comparing each sample to the unprocessed control (e.g., $\Delta L^{*}=L_{\mathrm{sample}}^{*}-L_{\mathrm{control}}^{*})$ [19]:

(6) $$\Delta E^{*}=\sqrt{\Delta L^{*2}+\Delta a^{*2}+\Delta b^{*2}}$$

The difference in perceivable color (ΔE*) between the unprocessed and processed peach–strawberry puree was interpreted based on the following classification: unnoticeable (ΔE = 0–0.5), slightly noticeable (ΔE = 0.5–1.5), noticeable (ΔE = 1.5–3.0), well visible (ΔE = 3.0–6.0) and great (ΔE = 6.0–12.0) [19].

The browning index (BI) was calculated according to Equation (7) as a common indicator of browning in food products [20]:

(7) $$\mathrm{BI}=\frac{180.232\left(a^{*}+1.75L^{*}\right)}{5.645L^{*}+a^{*}-3.012b^{*}}$$

### 2.8. Statistical Analysis

Data were expressed as mean ± standard deviation (SD). The statistical analysis was carried out using analysis of variance (ANOVA) and Tukey’s post hoc test was applied to evaluate significant differences among groups (*p* < 0.05).

### 2.9. Principal Component Analysis (PCA)

An Exploratory Factor Analysis (EFA) was used to uncover the underlying structure of the GC MS data set by SPSS Statistics 20 (IBM Software Group, Chicago, IL, USA) software. Data screening and extraction was performed by Principal Component Analysis (PCA) for assessing the main factor contribution in explaining the total variance, while the rotation method applied was Varimax. Data correlation and adequacy was assessed with Bartlett’s test of sphericity (significant for *p*-value < 0.05) and Kaiser normalization, considered acceptable when the value is ≥0.6. The number of principal components extracted was based on eigen values (higher than 1) and on the scree test (the position of the elbow in the eigen values plot) [21].

### 2.10. Sensorial Analysis of Peach–Strawberry Puree

Sensorial properties of the control and treated peach- strawberry purees were evaluated in the first day after processing, using a five-point Likert scale with boundary indications: 1—altered product, 5—very good product. The sensorial assessment included the following quality attributes: external appearance, color, flavor, taste, texture and aftertaste. A group of 25 panelists (18 women and 7 men in the age group 20–50) were trained according to ISO 6564:1985-E guidelines. Three-digit coded samples were provided to the panelists for evaluation 15 min after their removal from the chilled storage.

## 3. Results and Discussions

### 3.1. Comparative Effect of TT, HPP and HPMT on Microbial Load of Peach–Strawberry Puree

The assessment of the microbial stability evaluated the presence of spoilage microorganisms in the samples, immediately after treatments, after two weeks and after 21 days of refrigerated storage. The peach–strawberry puree is highly susceptible to spoilage due to its rich nutritive content, high free water content and relatively high initial microbial counts due to near ground growth of strawberries. The limited shelf-life of this fresh product could be extended by choosing effective treatments that should have minimal impact on the quality characteristics of the fruits. Evaluation of the effect of TT, HPP or HPMT upon the microbial load of peach–strawberry puree during 21 days of storage at 4 °C is presented in Table 1.

Considering the equivalence criteria, it can be noticed that TT and HPP have a similar effect on the MAB and M&Y population in the first day after the treatment, while HPMT displayed a higher inactivation rate of the MAB in the first day of storage. Consequently, in the first day of storage both TT (70 °C, 15 min) and HPP (600 MPa, 20 °C, 10 min) assured a 3-log bacterial reduction, while HPMT triggered a bacterial inactivation of approximately 5-log, with MAB values below the detection limit; all treatments inactivated the M&Y (approximately 4 log), reducing the counts below the detection limit.

For the control samples in the initial stage (day 1), similar values of MAB and M&Y population were obtained, of ~5.30 and ~4.70 log CFU·g^−1^ respectively, results which are in agreement with the reported values for control mango nectars and orange juice [22,23].

Two weeks of storage at 4 °C in limited oxygen environment (pouches) significantly reduced the initial MAB of both TT sample (less than one log reduction), and HPP treated sample (2-log bacterial inactivation). M&Y exhibited less than one log count for TT, HPP, HPMT.

At the end of the three weeks storage period at 4 °C, TT and HPP had a 2-log bacterial reduction compared to the initial day, while M&Y counts were below the detection limit from the beginning, in all samples (TT, HPP and HPMT). The control samples exhibited no significant changes in MAB and M&Y concentration during the 21 days of refrigerated storage.

Considering the microbial stability at the end of the storage period, all treated samples had similar MAB and M&Y values, indicating an equivalent inactivation effect of the applied treatments. All treated samples exhibited *Enterobacteriaceae* below the limit of detection (<1 log CFU·g^−1^) throughout the entire storage period (data not shown).

### 3.2. pH

Evaluation of pH changes in the untreated (control), TT, HPP and HPMT peach–strawberry purees is presented in Table 2. In the first day the HPP and HPMT treated peach–strawberry purees exhibited significantly lower pH values compared to the untreated peach–strawberry puree sample. The pH values significantly decreased in TT, HPP and HPMT peach–strawberry purees throughout the tested shelf- life period, more in the HPMT samples compared to the HPP ones. Similar pH variation was reported for strawberry purees treated by HPP (600 MPa, 3 min) [24].

### 3.3. Enzymatic Activity

#### 3.3.1. PPO

The residual activity of PPO showed that immediately after processing the highest reduction of the enzyme activity was achieved for the HPMT sample, followed by the TT sample, while in the case of HPP approximately a quarter of the initial activity was still present after the treatment. The authors of [7] reported a residual activity of PPO of approximately 55 to 60% after HPMT at 600 MPa and 40 °C after 1 to 5 min of treatment applied on pear slices, in the presence of sugar and citric acid. In another study performed by researchers [6] strawberry PPO was found to be highly resistant to HPMT at 600 MPa, 60 °C for 10 min, with a residual PPO activity in the processed samples of approximately 71.8% (600 MPa, 60 °C, 10 min). However, a study conducted on HPP peach pure indicated that PPO was susceptible to inactivation, after the HPMT treatment at 600 MPa, 50 °C for 10 min when the residual activity was of approximately 30% [8]. The differences reported in residual activity of PPO from fruits after high pressure and/or thermal treatments is influenced by fruit species and cultivars and in some cases, by the presence of latent enzymes that are released during processing [8,25].

**Figure 1:**
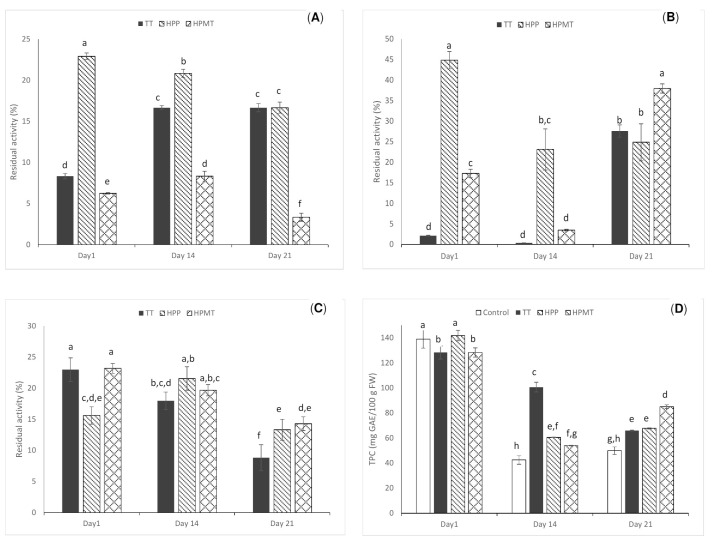
The stability of peach–strawberry puree TT (70 °C, 15 min), HPP (600 MPa, 10 min) and HPMT (600 MPa, 50 °C, 10 min) treated during refrigerated storage: (**A**) the residual activities of polyphenol oxidase (PPO); (**B**) the residual activities of peroxidase (POD); (**C**) the antiradical activity (DPPH) and (**D**) on the total polyphenol concentration (TPC). The error bars represent the standard deviation calculated on the average of three replicate samples. Different letters on each bar represent significant differences given by ANOVA and Tukey post hoc test (*p* < 0.05).

The current study found a more labile PPO compared to other studies performed in strawberry or peaches alone [8,25]. After two weeks of storage the activity of PPO in the TT sample was reactivated to a value two-fold higher than in the first day after the treatment. The PPO activity of the HPMT sample after two weeks of refrigerated storage was significantly higher than in the first day after the treatment (*p* < 0.05) while in the case of HPP sample, that displayed the highest PPO residual activity from all the analyzed samples, a slight decrease of the activity was registered within 14 days of storage. In the 21st day of refrigerated storage the TT and the HPP samples had equivalent residual activity (*p* > 0.05) while a decrease in the PPO activity to approximately 3% of the initial value was noticed for the HPMT sample (Figure 1A).

As it was previously reported [26] it is difficult to find a pattern of the PPO variation due to multiple factors influencing this enzyme activity. The enzymatic browning occurring during storage is attributed to PPO activity but it could also be influenced by pH, oxygen availability and temperature [27] and in this case a correlation of the rheological behavior, browning index but also the reduction in volatiles fingerprint could be linked with the PPO activity especially in the case of HPP sample where the highest residual PPO activity was noticed in the HPP samples on the first day of storage of approximately 23% and the RA values remained high until the end of storage.

#### 3.3.2. POD

The highest residual activity after treatments was displayed by POD and not by PPO and the same finding was reported by [28]. The pressure seemed to have a very low effect on POD, while the TT almost inactivated POD and approximately 44% of the residual activity was present in the HPMT samples and 17% in the HPP treated sample (Figure 1B). The authors of [25] found a residual POD activity ranging from 41.9% to 44.2% in strawberry puree treated at 600 MPa and 60 °C after 10 min. The same strong dependence of POD inactivation by temperature was reported in the study made by [7].

Interestingly the activity after 21 days of refrigerated storage, the residual POD activity increased in the TT samples and HPP samples and was similar (*p* > 0.05) for both treatments. The high activity of POD in the TT and HPP samples could also be related, in this case, with the synergistic effect of the PPO reaction products as substrates for POD reactions [6,29]. POD oxidizes phenols and other substrates, resulting in changes in color and the formation of off-flavors [30] and next to PPO contribute to the overall oxidative changes in fruit purees. The POD residual activity in the HPMT samples (*p* < 0.05) at the end of storage 40%, a value significantly higher (*p* < 0.05) than in the TT and HPP samples.

### 3.4. DPPH

The antiradical activity was comparable in the TT and HPMT samples in the first day of refrigerated storage and slightly lower in the HPP sample (*p* < 0.05). At the end of storage, the TT had the lowest antiradical activity while the HPMT and HPP had similar (*p* > 0.05) and slightly higher antiradical activity than the TT samples (*p* < 0.05), representing approximately 14% of the initial activity (Figure 1C). The authors of [31] studied the impact of HPP at 600 MPa for 5 min at 15 °C on aronia berry puree and concluded that the antiradical activity did not change significantly over 8 weeks of storage; however, the authors did not report the activity of PPO and POD. In the current study during 3 weeks of storage the antiradical activity was reduced in comparison with the first day of storage with 38% in TT samples, 62% in HPP samples and 85% in HPMT samples.

### 3.5. Total Phenolic Content

The phenolic content (TPC) reported in this study is in line with the values reported by other studies [32,33] in strawberry and other berry smoothie. In the first day of storage no significant differences were registered among fresh and HPP samples while a loss of approximately 8% was registered immediately after the HPMT and TT treatments (Figure 1D). A reduction of TPC with 9 to 24% in strawberry puree was reported by [6], in TT (88 °C/2 min) and HPP (600 MPa/20 °C/5 min) strawberries form different cultivars. The same research group reported no significant differences between the TT and the HPP samples and a reduction to a half of the initial concentration in TPC after 3 months of storage. The authors of [32] compared the strawberry smoothie TPC after TT (88 °C/2 min) and HPMT (500 MPa, 15 min, 50 °C) and reported 14% reduction after TT and 3% reduction after HPMT. Moreover, the reduction of TPC after the TT (85 °C, 5 min) of strawberry nectar was also reported in other research, for example, ref. [34] observed a 14% decrease in TPC.

In the current study, over the 3 weeks of storage the polyphenols content decreased in all samples. In the HPP and TT samples 50% of the total polyphenolic content was lost while in the HPMT sample only 33% of the initial content was lost. Enzymatic degradation of TPC could also be linked with PPO and POD activity and a 5-fold higher PPO residual activity was reported in the current study for the TT and HPP samples compared with HPMT samples; while for POD there was a 1.5-fold higher activity in the HPMT samples compared with HPP and TT samples in the 21st day of storage.

Non-enzymatic oxidation of TPC into quinones also could have contributed to degradation during storage. The authors of [6,32,35] found that the half-lives of TPC was between 350 and 170 days for TT (90 °C, 15 min) and the HPMT (500 MPa, 15 min, 50 °C), respectively. The authors of [36] reported after 30 days of refrigerated storage (4 °C) of strawberries flesh treated by TT (75 °C, 20 min) and after 400 MPa (5 min at 20 °C) an almost equal loss of polyphenols (25%) after both treatments, while [37] reported a 36% loss of TPC in strawberry puree stored for 11 weeks at 8° C. The authors of [38] reported at the end of the storage at 4 °C, significant losses (*p* < 0.05) of TPC in the untreated smoothie (about 15%), and in the HPP treated samples at 600 MPa for 3 min and 20 °C (11%), while in the smoothie treated by TT at 80 °C for 3 min, a decrease of 8% of TPC was indicated. After 21 days an equivalent concentration of TPC was present in the TT and HPT sample while HPMT had a significantly higher concentration (*p* > 0.05).

### 3.6. Rheology

**Figure 2:**
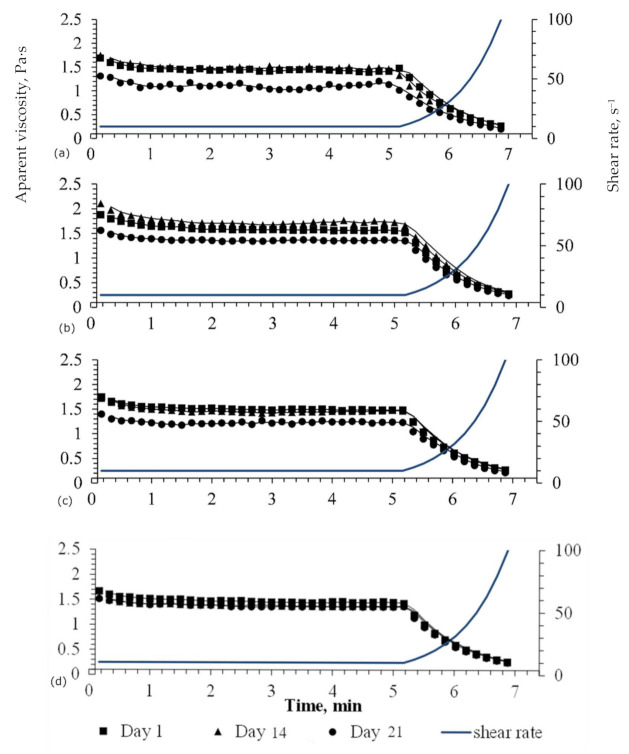
The effect of processing on rheological behavior of peach–strawberry puree. (**a**) Control; (**b**) TT (70 °C, 15 min); (**c**) HPP (600 MPa, 10 min); (**d**) HPMT (600 MPa, 50 °C, 10 min).

Peach–strawberry purees TT, HPP and HPMT treated were studied in terms of rheological behavior during 21 days of cold storage at 4 °C: in the first day after the treatments, after 1 week of storage and after two weeks of storage time in refrigeration conditions. Figure 2 shows the evolution of apparent viscosity as a function of the shear rate. The graphical presentation of the evolution of samples apparent viscosity with time and shear rate for different treatments was included in the Supplementary File Figure S2.

As a general trend, viscosity values during the steady shear ($\dot{\gamma }$ = 10 s^−1^; τ = 5 min) were mostly constant indicating a time independent behavior of the studied peach strawberry puree. The slightly decrease in viscosity values from the beginning of the test (during the first minute), from approx. 1.7 Pa∙s to 1.4 Pa∙s in control samples (Figure 1A), can be attributed to the inertia phenomenon of the steady state; with a pseudoplastic shear thinning behavior when increasing share rate from 10 to 100 s^−1^. The same behavior was registered in all treated samples.

It was also observed that during the entire period of storage the lowest viscosity values were recorded by the HPMT sample (Figure 2d). As indicated by [39], according to the principle of Le Chatelier, HPP determines a volume decrease of the proteins structure, the process being enhanced by TT, which affects both covalent and non-covalent bonds. The apparent viscosity, in this case, remained similar during the entire storage period (*p* > 0.05) for the HPMT samples that displayed the lowest initial viscosity value, while the other samples (TT, HPP and control) recorded a significant decrease of the viscosity after 21 days of storage (*p* < 0.05). The highest decrease in viscosity (*p* < 0.05) was registered for the control sample (Figure 2a) and this phenomenon could be associated with higher enzymatic activity in the control compared to the other samples [40]. An equivalent rheological behavior was displayed by the TT and HPP samples in the 21st day of storage and this could also be correlated with similar enzymatic (PPO and POD) activities in the samples (Figure 2b) that are explained by reactivation of enzymes during cold storage, noticed in other studies, as well [41,42].

In any case, results obtained in the present study confirms that HPMT by combining high pressure (600 MPa) with mild temperature values (50 °C) resulted in a weaker but better structural stability of the peach–strawberry puree attained during the 3 weeks of refrigerated storage (Figure 2d) and a similar rheological behavior of the HPP and TT samples after 21 days of refrigerated storage (Figure 2b,c).

### 3.7. The Volatile Profile

The fingerprints of VOCs identified in the control sample and the processed ones by TT, HPP and HPMT on the first and the 21st day of storage at 4 °C are indicated in Table 3.

GC analysis was performed in the first day and near the product’s shelf-life end, considering the relatively short shelf-life attributed to minimally processed fresh fruit purees and juices with no preservatives, in order to better evaluate the changes that occurred in the last part of product storage. This strategy enabled the assessment of the possibility to extend fruit purees’ shelf-life, in order to reduce waste and satisfy the interests of producers and consumers.

A total of 40 components were tentatively identified using NIST library including alcohols, aldehydes, esters, lactones and terpenoides. The compounds found in the current study were often reported by other authors in fruits, such as hexanal and other C6 derivates in strawberry purees [43] and peach [44,45], oleic acid, ionone, lactones in peach [46] and terpenoids in peach [44].

Hexanal has been associated with green and cut grass flavor characteristics in fruits [47]. A low decrease of the hexanal content was observed by [48] immediately after juice processing while in the current study a relatively high concentration of hexanal was found immediately after processing with a higher concentration in the TT samples compared to HPP and HPMT samples (*p* < 0.05). The increased concentration in all the processed samples could be due to increased release from the pulp after processing. However, after 21st days of cold storage, this compound could not be found in any of the analyzed sample. The authors of [49] also reported that the concentration of 2-hexanal severely declined during peach fruit cold storage over 21 days and so did aldehydes. It is assumed that the decrease in aldehydes is linked to the loss of freshness and a decrease in grassy and green notes of the fruits [43], and the same behavior was noticed for 1-methylciclohepthane with relatively high concentrations identified on the first day of storage in all samples. However, at the end of the storage, no concentration of this compound could have been identified (Table 2) in any of the analyzed samples. High concentrations of methyl esters were noticed in all samples at the end of storage with highest presence in the TT sample and the lowest in the HPP samples (*p* < 0.05). Methyl esters are formed in the presence of acyl-CoA and methanol [50]. From the total alcohols present in the peach–strawberry puree, heptatriacontanol had the highest concentration in the control sample. Triacontanol is a nontoxic growth stimulant for many plants. It plays an important role in plant development, and it is mainly present in strawberry, but its specific role in fruit development is still unclear (Pang et al., 2020). However, at the end of refrigerated storage this alcohol could not be identified in HMPT and TT samples, but it was present in low concentration in the HPP sample (*p* > 0.05). Alternatively, high concentrations of iso-heptatriacontanol could be found in the 21st day of storage in all treated samples and higher concentrations was found in the HPP samples compared to HPMT and TT samples (*p* < 0.05). The ethyl ketone derivate with the C_17_H_26_O_2_ formula also called vetiveryl acetate was associated mainly with raspberries and with fruity notes [51]. Although its concentration decreased over storage, non-significant differences (*p* > 0.05) could be reported between all the samples analyzed in on the 21st day of storage. From the total number of the five lactones identified, γ and δ-lactones were the most abundant. The highest concentration of total lactones was found in the control sample followed by the HPP and HPMT samples while in the TT samples the lowest concentration was present (*p*< 0.05) (Table 2). Over time the concentration of lactones was constant in the control sample, while the highest concentration was present in the HPP sample (PEG-lactone) in the 21st day of storage. Microorganisms could have metabolized the lactones present during the first days of storage in the HPP and HPMT samples to enhance their survival rate until the substrate was completely spent [52]. Interestingly, the most abundant terpenoids concentrations were found in the TT samples in the first day of storage, however the concentration at the end of storage is almost equal (*p* > 0.05) in all the samples.

**Figure 3:**
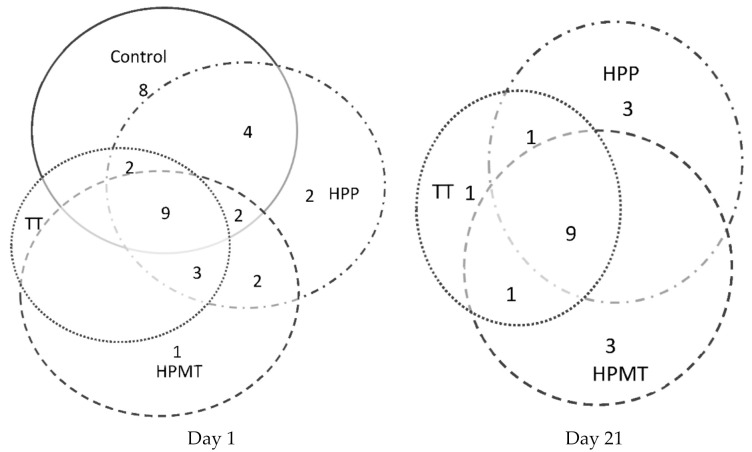
Venn diagram for the volatile compounds’ numbers detected by SPME/GC-MS (1st and 21st day of storage) TT (70 °C, 15 min), HPP (600 MPa, 10 min) and HPMT (600 MPa, 50 °C, 10 min).

When analyzing the total distribution of volatiles in the first day, HPP and control samples had the highest number of volatile compounds, followed by HPMT while the lowest number of volatile compounds was found in the TT samples as shown in the Venn diagram (Figure 3).

In the last day of storage, a reduced number of components compared with the first day, but similar number of compounds in all samples were found in the HPP, HPMT and TT samples, indicating that storage somehow equalized the differences in volatiles profile between the samples and suggesting that deleterious process are continuing even during cold storage of the packed products. This similarity of the volatile profile could be explained for the TT and HPP samples by the high residual PPO activity in the 21st day of storage, while for the HPMT samples by the high POD residual activity (Figure 1).

**Figure 4:**
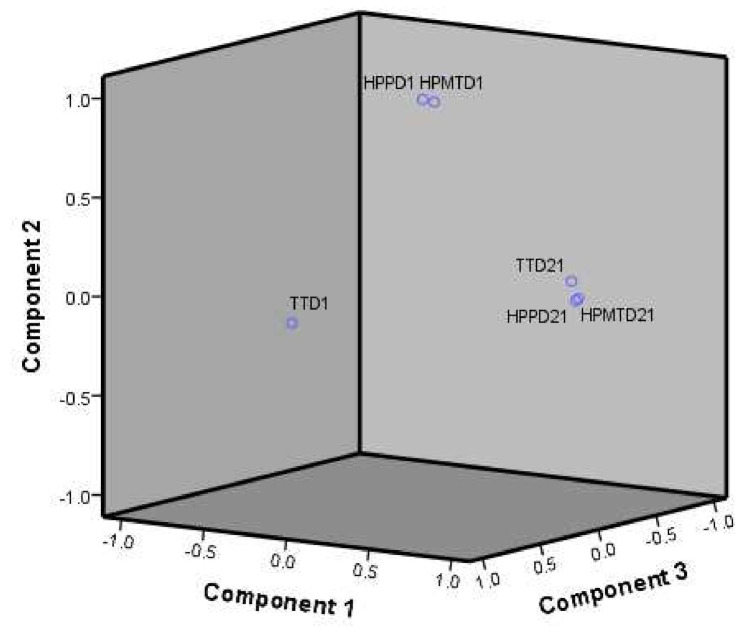
The 3D plot of the principal components (PCs) and their internal structure resulted from EFA, with Varimax rotation.

The PCA of the volatiles in all the samples shows that on the first day the samples were different and while HPP and HPMT were close to each other and closer to control, the TT sample displayed a different fingerprint of volatiles. In the last day of storage, the volatile profile of the HPP, HPMT and TT samples is almost overlapped and belonging to the same cluster (Figure 4) indicating an equivalency in the volatile profile at the end of storage.

### 3.8. Changes in Color Attributes

The color changes throughout 21 days of peach–strawberry purées storage in TT, HPP and HPMT samples are illustrated in Table 3. The color of strawberry-based products is mostly determined by anthocyanins concentrations and type, which is a cultivar dependent trait [53]. During storage several non-enzymatic reactions can take place, resulting in color degradation of the strawberry-based product: anthocyanin degradation and phenol polymerization, ascorbic acid degradation, Maillard reactions and/or sugar degradation acid catalyzed. Several studies indicate that TT of strawberry-based products resulted in substantial loss of anthocyanins [29,53,54]. The change of a* values across different treatments indicated that the redness of the samples is significant (*p* < 0.05) for both samples thermally treated (TT and HPMT), while HPP samples did not display a significant change of color compared to the raw sample for the entire refrigerated storage period. Coordinates b*, indicating the yellowish color of the samples was 1.8-fold higher (*p* < 0.05) for the TT samples compared to all other samples, most probably due to the non-enzymatic browning. Moreover, HPP and HPMT samples had b* values with no significant differences (*p* > 0.05) compared to the control (raw) samples, which could be an argument for the hypothesis that HPP does not promote Maillard reactions, responsible for sample browning. HPP determined the lowest difference in color (ΔE) of all treatments: the initial ΔE_1_* of 0.76 ± 0.18 designates a slightly noticeable change in color; the values recorded after 14 and 21 days of storage (ΔE_14_* = 2.25 ± 0.24; ΔE_21_* = 2.21 ± 0.27) are similar (*p* > 0.05) indicating a significant change of color (*p* < 0.05) only compared to day 1. Similar values of ΔE* were reported for HPP strawberry puree at 600 MPa (3 min) [24]. The TT led to the highest change of color (*p* < 0.05) compared with the other samples; however, no significant changes (*p* > 0.05) could be observed throughout the 21 days of storage. The combined HPMT treatment was responsible for a well visible change of color just after the treatment, followed by a great change of color compared to the control after 14 and 21 days of storage, according to the classification of color differences indicated by [19]. The higher chroma (C*) values for TT samples indicate a significantly higher intensity (*p* < 0.05) of the color as perceived by the human eye than for the other samples: with 15.8% higher than HPP sample and 23.6% higher than HPMT samples in the first day of storage and similar differences in C-values registered through the 21 days of refrigerated storage.

The hue angles (h*) of approx. 30° for all samples except the TT indicate a red hue. The 59.29 ± 0.51° hue angle of TT samples after 14 days of storage indicates a change of color from red to yellow tones, which could be attributed to the formation of brown pigments [53].

**Figure 5:**
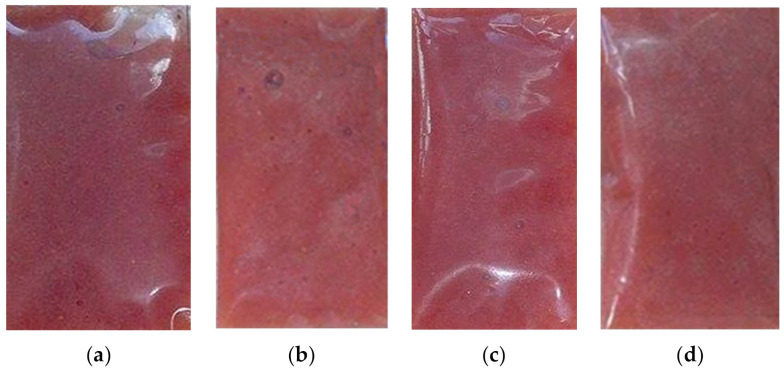
Color estimation of (**a**) control (raw); (**b**) TT; (**c**) HPP; (**d**) HPMT sample.

The browning indexes (BI) of TT sample (1st day) is 1.5 higher than that of the control sample and confirms the presence of brown pigments. The HPP sample has similar BI value to the control, while HPMT samples had BI 0.87 times lower compared to the control samples (1st day). The reduced PPO activity (3% residual activity) in the HPMT sample could justify a higher BI index for the HPP samples (16.6% residual activity) compared to HPMT sample, in the 21st day of storage (Figure 5A). The complexity of color parameters made hard to establish equivalency criteria among the TT, HPP and HPMT treatments when judging the values presented in Table 3. Accordingly, when considering the BI values in the 1st day and the 21st day of storage there are significant differences (*p* < 0.05) between the TT, HPP and HPMT samples.

### 3.9. Sensory Profile of Peach–Strawberry Puree

**Figure 6:**
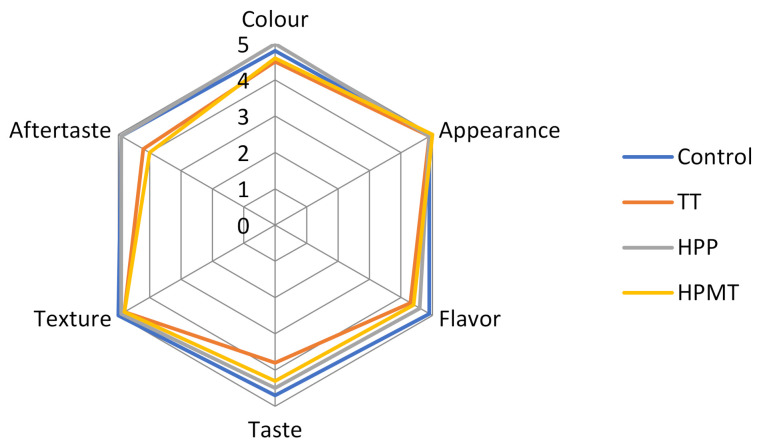
Sensory profile of peach–strawberry puree, representing the mean values of the individual points given by the 25 panellists for the samples.

Sensory profiling of peach–strawberry purees showed that there were differences between the blank and TT, HPP and HPMT purées samples (Figure 6). The unprocessed sample is the best one compared to all three processed samples (HPP, TT and HPMT) in terms of taste, flavor, texture and aftertaste. However, the panelists have appreciated more the color of HPP sample than all the other samples. This observation is in agreement with the browning index determined with the colorimetric analysis (Table 4). The lowest scores in terms of taste and flavor have been acquired by the TT sample and there result is confirmed by the GC/Ms analysis (Table 2) and the Venn diagram (Figure 3). However, the appearance of the peach–strawberry purees was the same for consumers who did not discriminate differences in texture properties among samples.

## 4. Conclusions

This study shows that the dynamics of the microbial and quality changes and the interrelated parameters makes equivalent effects to be present at different time points in the TT, HPP and HPMT treated samples during refrigerated storage.

- In the first day of storage TT and HPP samples had equivalent microbial stability, while after 21st day of refrigerated storage all treatments (TT, HPP and HPMT) could be considered equivalent.
- The rheology of the peach–strawberry puree after 21 days of refrigerated storage could be considered equivalent for the TT and HPP treatments.
- As expected, the HPP treated sample had the most appreciated sensorial profile compared to HPMT and TT samples.
- No equivalency could be considered regarding color for TT, HPP and HPMT samples during storage period.
- The volatile profile of peach–strawberry puree showed equivalence for the HPP and HPMT samples in the first day of storage, while after 21 days of refrigerated storage all the treated samples (TT, HPP and HPMT) could be considered equivalent.
- The PPO and POD enzymes inactivation indicated an equivalent effect for the TT and HPP samples in the 21st day of storage and the same conclusion applies to TPC content.
- The antiradical activity indicates an equivalence between HPP and HPMT samples after 21 days of storage.
- Further studies should be focused on safety criteria equivalence considering target pathogens (i.e., *Salmonella*) in the same processing conditions as identified in the present study.

## Figures and Tables

**Table 1:** Microbial counts changes for TT, HPP and HPMT peach–strawberry purees during 21 days of refrigerated storage.

Treatment	Storage Period (Days)	MAB (Log CFU g^−1^)	M&Y (Log CFU g^−1^)
Control (raw peach–strawberry puree)	1	5.33 ± 0.05 *^,a^	4.60 ± 0.01 ^a^
	14	5.82 ± 0.03 ^a^	4.76 ± 0.01 ^a^
	21	6.32 ± 0.04 ^a^	5.06 ± 0.06 ^a^
TT	1	2.17 ± 0.06 ^b^	<1.00 ^b^
(70 °C, 15 min)	14	1.85 ± 0.02 ^c^	<1.00 ^b^
	21	<1.00 ^d^	<1.00 ^b^
HPP (600 MPa, 20 °C, 10 min)	1	2.07 ± 0.07 ^b,c^	<1.00 ^b^
	14	<1.00 ^d^	<1.00 ^b^
	21	<1.00 ^d^	<1.00 ^b^
HPMT	1	<1.00 ^d^	<1.00 ^b^
(600 MPa, 50 °C, 10 min)	14	<1.00 ^d^	<1.00 ^b^
	21	<1.00 ^d^	<1.00 ^b^

* ± standard deviation; rows with different superscripts are significantly different (*p* < 0.05) by Tukey test; MAB = mesophilic aerobic bacteria.

**Table 2:** Variation of pH in peach–strawberry purees during refrigerated storage.

	Control	TT (70 °C, 15 min)	HPP (600 MPa, 10 min)	HPMT (600 MPa, 50 °C, 10 min)
Day 1	3.72 ± 0.01 ^a^	3.71 ± 0.01 ^a,b^	3.61 ± 0.01 ^a,b,c^	3.62 ± 0.01 ^a,b,c^
Day 14	3.66 ± 0.01 ^a,b,c^	3.42 ± 0.03 ^e,f^	3.57 ± 0.02 ^b,c,d^	3.37 ± 0.08 ^f^
Day 21	3.70 ± 0.10 ^a,b^	3.45 ± 0.02 ^d,e,f^	3.54 ± 0.06 ^c,d,e^	3.37 ± 0.02 ^f^

± standard deviation; rows with different superscripts are significantly different (*p* < 0.05) by Tukey test.

**Table 3:** The GC/MS SPME volatiles concentration (μL/kg octanol) in control sample on the first day of storage and TT, HPP and HPMT samples in the first and 21st day of storage at 4 °C.

Components	KI	Ion Fragments	Class	Control D1	HPP D1	HPP D21	HPMT D1	HPMT D21	TT D1	TT D21
2-Methyl-1-pentene	886	55; 56; 67; 69	AlHc	00.00 ± 0.00 ^c^	0.56 ± 0.07 ^b^	0.00 ± 0.00 ^c^	1.25 ± 0.13 ^a^	0.00 ± 0.00 ^c^	0.00 ± 0.00 ^c^	0.00 ± 0.00 ^c^
Cycloheptadiene	887	102; 103; 104; 105	AlHc	2.13 ± 0.06 ^a^	0.00 ± 0.00 ^b^	0.00 ± 0.00 ^b^	0.00 ± 0.00 ^b^	0.00 ± 0.00 ^b^	0.00 ± 0.00 ^b^	0.00 ± 0.00 ^b^
3,3-Dimethyl-1-hexene	904	55; 56; 67; 69	AlHc	7.27 ± 0.09 ^b,c^	2.46 ± 0.22 ^d^	2.77 ± 0.31 ^c,d^	3.32 ± 0.21 ^c,d^	2.77 ± 0.19 ^c,d^	44.71 ± 5.71 ^a^	2.86 ± 0.22 ^c,d^
z,z,z-4,6,9-Nonadecatriene	943	77; 79; 91; 93	AlHc	4.50 ± 0.05 ^a^	5.49 ± 0.39 ^b^	8.26 ± 0.55 ^b^	7.54 ± 0.59 ^b^	7.54 ± 0.77 ^b^	23.13 ± 1.54 ^a^	23.13 ± 2.21 ^b,c^
n-Dotriacontane	953	57; 71; 73; 85	AlHc	1.39 ± 0.01 ^c^	0.55 ± 0.03 ^e^	0.85 ± 0.10 ^d,e^	1.99 ± 0.17 ^b^	1.23 ± 0.13 ^c,d^	3.49 ± 0.39 ^a^	1.67 ± 0.12 ^b,c^
2 Methyl cyclopentanol	837	53; 55; 57; 67	Alcohol	0.00 ± 0.00 ^b^	0.00 ± 0.00 ^b^	0.00 ± 0.00 ^b^	1.42 ± 0.12 ^a^	0.00 ± 0.00 ^b^	0.00 ± 0.00 ^b^	0.00 ± 0.00 ^b^
Tri(2-etilbutanol)glycerol	874	55; 73; 87; 99	Alcohol	0.00 ± 0.00 ^b^	0.00 ± 0.00 ^b^	0.00 ± 0.00 ^b^	0.00 ± 0.00 ^b^	0.00 ± 0.00 ^b^	0.00 ± 0.00 ^b^	0.00 ± 0.00 ^b^
4-Methyl-3-pentene-2-ol	902	65; 66; 71; 82	Alcohol	0.00 ± 0.00 ^b^	1.43 ± 0.09 ^b^	2.35 ± 0.19 ^b^	2.63 ± 0.25 ^b^	3.57 ± 0.37 ^b^	76.63 ± 6.59 ^a^	1.89 ± 0.09 ^b^
10-Undecyne-1-ol	926	55; 65; 67; 77	Alcohol	0.00 ± 0.00 ^b^	0.88 ± 0.07 ^a^	0.00 ± 0.00 ^b^	0.79 ± 0.08 ^a^	0.00 ± 0.00 ^b^	0.00 ± 0.00 ^b^	0.00 ± 0.00 ^b^
1-Heptatriacotanol	1080	85; 95; 110; 111	Alcohol	18.39 ± 1.22 ^b^	15.84 ± 1.72 ^b^	1.01 ± 0.01 ^c^	0.00 ± 0.00 ^c^	0.00 ± 0.00 ^c^	16.33 ± 1.25 ^b^	0.00 ± 0.00 ^c^
Iso-heptatriacotanol	1082	57; 67; 85; 95	Alcohol	0.00 ± 0.00 ^c^	0.00 ± 0.00 ^c^	16.62 ± 1.85 ^a^	0.00 ± 0.00 ^c^	13.75 ± 1.55 ^b^	0.00 ± 0.00 ^c^	13.75 ± 1.33 ^b^
1,1 Dimethylethyl phenol	1119	57; 67; 81; 91	Alcohol	2.29 ± 0.29 ^a^	1.46 ± 0.15 ^b^	2.66 ± 0.28 ^a^	0.00 ± 0.00 ^c^	0.00 ± 0.00 ^c^	0.00 ± 0.00 ^c^	0.00 ± 0.00 ^c^
2 Methylene-(3 a,5 a) cholesten 3-ol	1122	67; 79; 83; 95	Alcohol	1.56 ± 0.16 ^a^	0.43 ± 0.03 ^c^	0.00 ± 0.00 ^d^	0.00 ± 0.00 ^d^	0.00 ± 0.00 ^d^	0.00 ± 0.00 ^d^	2.91 ± 0.31 ^a^
2-Hexenal	876	55; 61; 69; 83	Aldehydes	9.49 ± 0.94 ^c^	22.50 ± 2.23 ^b^	0.00 ± 0.00 ^d^	21.94 ± 2.18 ^b^	0.00 ± 0.00 ^d^	78.78 ± 7.28 ^a^	0.00 ± 0.00 ^d^
1-Methylcycloheptane	892	55; 57; 58; 59	Aldehydes	0.00 ± 0.00 ^b^	1.28 ± 0.13 ^b^	0.00 ± 0.00 ^b^	3.52 ± 0.34 ^b^	0.00 ± 0.00 ^b^	23.247 ± 7.77 ^a^	0.00 ± 0.00 ^b^
Dione	922	69; 97; 113; 129	Dione	0.00 ± 0.00 ^b^	0.00 ± 0.00 ^b^	0.00 ± 0.00 ^b^	0.00 ± 0.00 ^b^	0.00 ± 0.00 ^b^	0.00 ± 0.00 ^b^	0.00 ± 0.00 ^b^
Methyl ester derivate	1038	53; 55; 67; 79	Ester	8.66 ± 0.72 ^c^	3.31 ± 0.32 ^d^	10.32 ± 1.05 ^a^	0.00 ± 0.00 ^e^	11.52 ± 1.13 ^b^	2.51 ± 0.23 ^e^	14.85 ± 1.42 ^a^
Methyl decanoate	1074	50; 51; 76; 77	Ester	0.00 ± 0.00 ^c^	1.13 ± 0.10 ^b,c^	2.47 ± 0.22 ^b^	0.00 ± 0.00 ^c^	0.00 ± 0.00 ^b^	0.00 ± 0.00 ^c^	1.29 ± 0.27 ^b,c^
Derivate of methyl arachidonate	1094	55; 67; 81; 95	Ester	3.31 ± 0.41 ^b^	3.31 ± 0.34 ^b^	0.00 ± 0.00 ^a^	3.31 ± 0.31 ^b^	0.00 ± 0.00 ^a^	3.31 ± 0.35 ^b^	0.00 ± 0.00 ^a^
2 Luamin	1020	51; 55; 57; 69	Ether	0.89 ± 0.07 ^a^	0.00 ± 0.00 ^b^	0.00 ± 0.00 ^b^	0.00 ± 0.00 ^b^	0.00 ± 0.00 ^b^	0.00 ± 0.00 ^b^	0.00 ± 0.00 ^b^
1 Mono linolenin	1103	55; 67; 81; 95	Fatty acids	0.44 ± 0.05 ^c^	0.00 ± 0.00 ^d^	0.00 ± 0.00 ^d^	0.00 ± 0.00 ^d^	1.92 ± 0.15 ^b^	0.00 ± 0.00 ^d^	0.00 ± 0.00 ^d^
Oleic acid	932	55; 57; 65; 67	Fatty acids	0.00 ± 0.00 ^a^	0.00 ± 0.00 ^a^	0.00 ± 0.00 ^a^	0.00 ± 0.00 ^a^	0.92 ± 0.08 ^b^	0.00 ± 0.00 ^a^	0.00 ± 0.00 ^a^
Rutin	1026	70; 81; 100; 101	Flavonoides	4.43 ± 0.42 ^a^	1.42 ± 0.16 ^b^	0.00 ± 0.00 ^c^	1.42 ± 0.11 ^b^	0.00 ± 0.00 ^c^	0.00 ± 0.00 ^c^	0.00 ± 0.00 ^c^
Styrene	885	56; 78; 79; 103	ArHc	0.00 ± 0.00 ^a^	0.00 ± 0.00 ^a^	0.00 ± 0.00 ^a^	0.00 ± 0.00 ^a^	0.00 ± 0.00 ^a^	0.00 ± 0.00 ^a^	0.00 ± 0.00 ^a^
Ethyl ketone derivate	1014	57; 81; 91; 119	Ketones	24.64 ± 2.28 ^b^	5.19 ± 0.53 ^d^	10.09 ± 1.05 ^c,d^	5.70 ± 0.46 ^d^	8.12 ± 0.79 ^c,d^	5.39 ± 0.54 ^d^	8.93 ± 0.78 ^c,d^
PEG-Lactone	1051	121; 135; 149; 150	Lactone	0.00 ± 0.00 ^a^	0.00 ± 0.00 ^a^	8.65 ± 0.84 ^b^	0.00 ± 0.00 ^a^	0.00 ± 0.00 ^a^	0.00 ± 0.00 ^a^	0.00 ± 0.00 ^a^
γ-Lactone	1062	51; 55; 67; 81	Lactone	5.07 ± 0.52 ^a^	0.00 ± 0.00 ^b^	0.00 ± 0.00 ^b^	0.00 ± 0.00 ^b^	0.00 ± 0.00 ^b^	0.00 ± 0.00 ^b^	0.00 ± 0.00 ^b^
δ-Heptalactone	1066	55; 67; 79; 81	Lactone	2.27 ± 0.21 ^a^	0.40 ± 0.03 ^c^	0.00 ± 0.00 ^d^	0.00 ± 0.00 ^d^	0.00 ± 0.00 ^d^	0.00 ± 0.00 ^d^	0.00 ± 0.00 ^d^
10-Methyl-8-tetradecen-1-ol acetate	1077	55; 67; 81; 95	Lactone	15.15 ± 1.12 ^a^	0.00 ± 0.00 ^b^	0.00 ± 0.00 ^b^	0.00 ± 0.00 ^b^	0.00 ± 0.00 ^b^	0.00 ± 0.00 ^b^	0.00 ± 0.00 ^b^
γ-Lactone	1088	55; 57; 67; 81	Lactone	2.41 ± 0.22 ^a^	2.42 ± 0.41 ^a^	0.00 ± 0.00 ^b^	2.41 ± 0.41 ^a^	0.00 ± 0.00 ^b^	0.00 ± 0.00 ^b^	0.00 ± 0.00 ^b^
Campesterol	1072	55; 67; 83; 97	Sterols	0.00 ± 0.00 ^a^	0.90 ± 0.07 ^b^	0.00 ± 0.00 ^a^	0.00 ± 0.00 ^a^	0.00 ± 0.00 ^a^	0.00 ± 0.00 ^a^	0.00 ± 0.00 ^a^
Oblongifoliol	1059	51; 55; 67; 81	Terpenoids	4.76 ± 0.41 ^a^	0.30 ± 0.02 ^b,c^	0.79 ± 0.77 ^b,c^	0.00 ± 0.00 ^c^	0.93 ± 0.07 ^b,c^	0.00 ± 0.00 ^c^	1.09 ± 0.08 ^b^
6-Methyl-c-ionone	1041	55; 77; 79; 91	Terpenoids	6.56 ± 0.54 ^a^	0.00 ± 0.00 ^b^	0.00 ± 0.00 ^b^	0.00 ± 0.00 ^b^	0.00 ± 0.00 ^b^	0.00 ± 0.00 ^b^	0.00 ± 0.00 ^b^
Curcumol	986	55; 67; 83; 95	Terpenoids	1.33 ± 0.12 ^b^	1.62 ± 0.18 ^a^	0.00 ± 0.00 ^c^	1.28 ± 0.12 ^b^	0.00 ± 0.00 ^c^	1.28 ± 0.14 ^b^	0.00 ± 0.00 ^c^
Isochiapin B	989	53; 55; 57; 71	Terpenoids	2.47 ± 0.25 ^b^	1.60 ± 0.19 ^c^	0.00 ± 0.00 ^d^	0.31 ± 0.02 ^d^	3.93 ± 0.04 ^a^	0.31 ± 0.03 ^d^	0.00 ± 0.00 ^d^
Coniferyl aldehyde	1012	57; 76; 104; 121	Terpenoids	28.34 ± 2.26 ^a^	0.00 ± 0.00 ^b^	0.00 ± 0.00 ^b^	0.00 ± 0.00 ^b^	0.00 ± 0.00 ^b^	0.00 ± 0.00 ^b^	0.00 ± 0.00 ^b^
Ambroxide	1022	55; 67; 69; 81	Terpenoids	0.00 ± 0.00 ^a^	1.37 ± 0.14 ^a^	0.00 ± 0.00 ^a^	1.41 ± 0.13 ^a^	0.00 ± 0.00 ^a^	88.83 ± 7.99 ^b^	0.00 ± 0.00 ^a^
Gemacrene derivate	1071	53; 55; 57; 67	Terpenoids	5.62 ± 0.51 ^a^	0.00 ± 0.00 ^b^	0.00 ± 0.00 ^b^	0.00 ± 0.00 ^b^	0.00 ± 0.00 ^b^	0.00 ± 0.00 ^b^	0.00 ± 0.00 ^b^
δ-Cadinol	983	55; 57; 79; 91	Terpenoids	4.44 ± 0.50 ^d,e^	2.89 ± 0.21 ^c^	11.45 ± 0.14 ^e^	4.54 ± 0.48 ^d^	12.42 ± 0.13 ^b,c^	5.79 ± 0.59 ^d^	15.11 ± 0.18 ^a^
1-Methyl-3-(1-methylethyl)-benzene	890	91; 92; 111; 115	ArHc	0.00 ± 0.00 ^a^	0.00 ± 0.00 ^a^	0.00 ± 0.00 ^a^	0.00 ± 0.00 ^a^	1.19 ± 0.12 ^b^	0.00 ± 0.00 ^a^	1.45 ± 0.15 ^b^

TT—thermally treated samples (70 °C, 15 min), HPP—high pressure processed samples (600 MPa, 10 min) and HPMT-high pressure mild thermally treated samples (600 MPa, 50 °C, 15 min). AlHc—aliphatic hydrocarbons, ArHc—aromatic hydrocarbons. Different letters indicate significant differences (*p* < 0.05) on each row by post hoc Tuckey test; KI—Kovats Index.

**Table 4:** Color changes in samples compared to control during 21 days of refrigerated storage.

	Control						TT (70 °C, 15 min)							HPP (600 MPa, 20 °C,10 min)							HPMT (600 MPa, 50 °C,10 min)						
	L*	a*	b*	C*	h°	BI	L*	a*	b*	ΔE*	C*	h°	BI	L*	a*	b*	ΔE*	C*	h°	BI	L*	a*	b*	ΔE*	C*	h°	BI
Day 1	41.52 ± 0.85 ^b,A,^*	33.10 ± 0.63 ^b,B^	18.58 ± 0.48 ^a,A^	38.52 ± 0.32 ^b,B^	28.95 ± 1.05 ^a,A^	110.25 ± 3.54 ^a,B^	46.03 ± 0.07 ^a,B^	29.52 ± 0.17 ^b,A^	34.52 ± 0.22 ^a,C^	17.17 ± 0.67 ^a,C^	45.48 ± 0.06 ^b,C^	49.84 ± 0.36 ^a,C^	168.34 ± 1.43 ^a, C^	42.32 ± 0.11 ^b,A^	32.90 ± 0.15 ^b,B^	19.50 ± 0.15 ^a,B^	0.76 ± 0.18 ^a,A^	38.30 ± 0.05 ^a,B^	30.32 ± 0.22 ^a,A^	112.86 ± 0.09 ^a,B^	45.44 ± 0.20 ^a,B^	29.01 ± 0.26 ^a,A^	18.72 ± 0.04 ^a,A,B^	5.43 ± 0.25 ^a,B^	34.73 ± 0.20 ^a,A^	33.01 ± 0.01 ^b,B^	96.94 ± 0.68 ^a,A^
Day 14	38.61 ± 0.33 ^a,A^	31.78 ± 0.39 ^a,A^	18.99 ± 0.13 ^a,A^	36.68 ± 0.27 ^a,A^	31.17 ± 0.63 ^a,A^	121.80 ± 1.22 ^b,A^	41.43 ± 0.20 ^b,B^	20.66 ± 0.10 ^a,B^	34.04 ± 0.11 ^a,B^	19.13 ± 0.12 ^a,A^	39.89 ± 0.07 ^a,B^	59.29 ± 0.51 ^a,B^	178.04 ± 0.14 ^b,B^	40.42 ± 0.19 ^a,C^	32.41 ± 0.09 ^a,A^	19.48 ± 0.09 ^a,A^	2.25 ± 0.24 ^b,B^	37.19 ± 0.62 ^a,A^	30.98 ± 0.02 ^a,A^	115.96 ± 2.20 ^a,C^	45.21 ± 0.02 ^a,D^	31.19 ± 0.00 ^b,A^	18.79 ± 0.13 ^a,A^	7.27 ± 0.65 ^b,C^	36.33 ± 0.08 ^b,A^	31.18 ± 0.22 ^a,A^	99.09 ± 0.91 ^a,D^
Day 21	39.65 ± 0.11 ^a,A^	32.77 ± 0.31 ^a,b,A^	18.64 ± 0.34 ^a,A^	37.43 ± 0.10 ^a,A^	29.88 ± 0.63 ^a,A^	117.36 ± 1.46 ^b,A^	42.81 ± 0.17 ^b,B^	29.08 ± 0.11 ^a,b,B^	33.83 ± 0.43 ^a,B^	15.43 ± 0.84 ^a,A^	44.37 ± 0.23 ^a,b,B^	48.87 ± 0.37 ^a,B^	179.71 ± 0.25 ^b,B^	41.22 ± 0.09 ^a,b, C^	32.57 ± 0.16 ^a,b,A^	19.42 ± 0.18 ^a,A^	2.21 ± 0.27 ^b,B^	37.82 ± 0.10 ^a,A^	30.53 ± 0.43 ^a,A^	113.67 ± 1.87 ^a,C^	46.80 ± 0.29 ^b,D^	31.62 ± 0.04 ^b,C^	18.76 ± 0.25 ^a,A^	7.38 ± 0.12 ^b,C^	36.45 ± 0.32 ^b,C^	30.32 ± 0.22 ^a,A^	95.25 ± 1.43 ^a,D^

* Different letters indicate significant differences (*p* < 0.05) among days (small caps) and sample treatments (capital letters) by post hoc Tukey test.

## Data Availability

Not applicable.

## Supplementary Materials

The following are available online at https://www.mdpi.com/article/10.3390/foods10112580/s1, Figure S1: Pressure—temperature profile of the (A) HPP (600 MPa, 20 °C, 10 min) and (B) HPMT (600 MPa, 50 °C, 10 min). Temperature was measured by a temperature probe placed in the vessels in the surrounding liquid, Figure S2: Evolution of peach-strawberry puree appare.nt viscosity with time and shear rate for TT (70 °C, 15 min), HPP (600 MPa, 10 min) and HPMT (600 MPa, 50 °C, 10 min).
