# Supplementary material for: Quality and Stability Equivalence of High Pressure and/or Thermal Treatments in Peach–Strawberry Puree. A Multicriteria Study
Source: Foods. 2021 Oct 26;10(11):2580. doi: 10.3390/foods10112580 (PMC8622699; doi:10.3390/foods10112580)
Supplement: Supplementary file 1 [file foods-10-02580-s001.zip › Supplementary _Figures Captions.pdf]

Supplementary figures captions

Figure 1_Supplementary file Pressure - temperature profile of the A) HPP (600 MPa, 20°C, 10 min) and B) HPMT (600 MPa, 50°C, 10 min). Temperature was measured by a temperature probe placed in the vessels in the surrounding liquid

Figure 2_Supplementary file. Evolution of peach-strawberry puree apparent viscosity with time and shear rate for TT (70°C, 15 min), HPP (600MPa, 10 min) and HPMT (600 MPa, 50°C, 10 min)
