# Supplementary figures and images for: Quality and Stability Equivalence of High Pressure and/or Thermal Treatments in Peach–Strawberry Puree. A Multicriteria Study
Source: Foods. 2021 Oct 26;10(11):2580. doi: 10.3390/foods10112580 (PMC8622699; doi:10.3390/foods10112580)

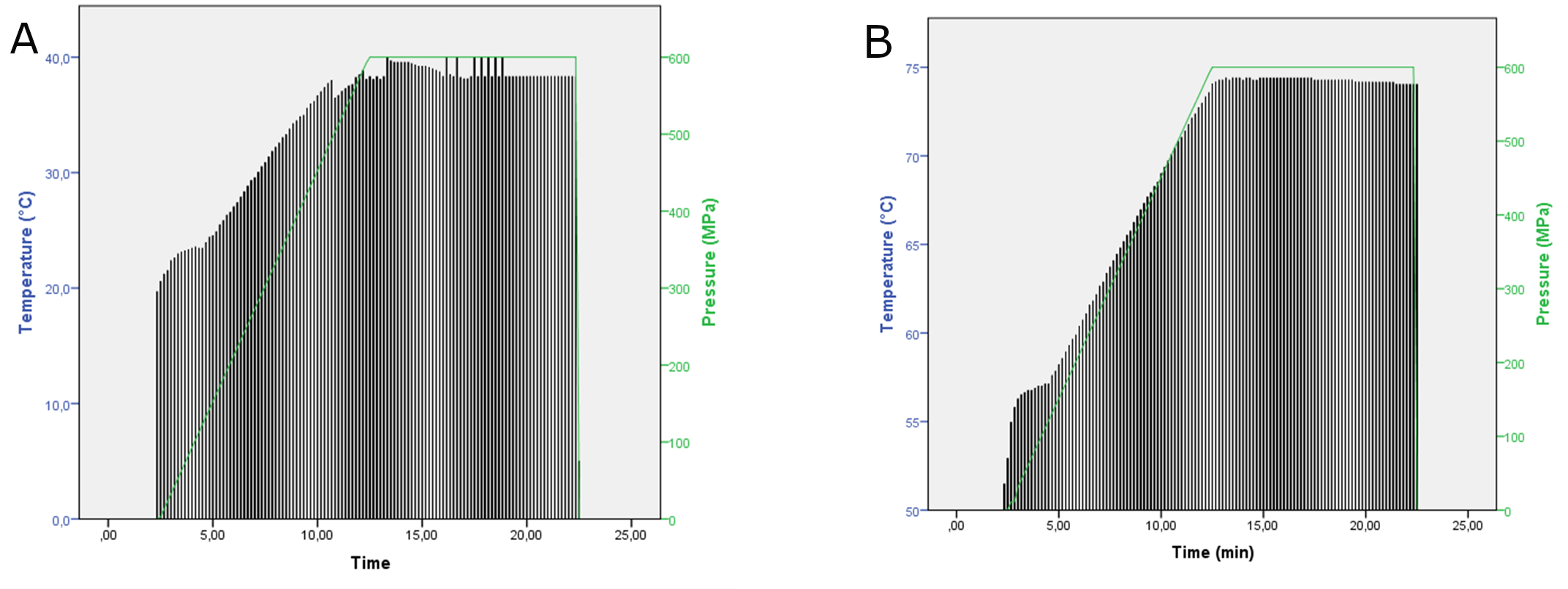

Supplement: Supplementary file 1 [file foods-10-02580-s001.zip › Supplementary file_Figure 1_Pressure- temperature profile HPP, HPMT.tif]

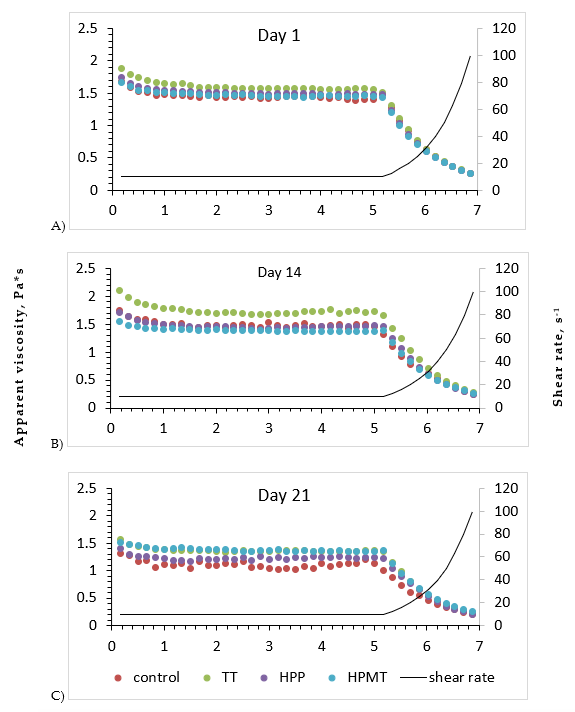

Supplement: Supplementary file 1 [file foods-10-02580-s001.zip › Supplementary file_Figure 2_Apparent viscosity, shear rate.tif]
